# Commentary—fat but fit…and cold? Potential evolutionary and environmental drivers of metabolically healthy obesity

**DOI:** 10.1093/emph/eoac030

**Published:** 2022-08-16

**Authors:** Cara Ocobock, Alexandra Niclou

**Affiliations:** Department of Anthropology, University of Notre Dame, Notre Dame, IN, USA; Eck Institute for Global Health, Institute for Educational Initiatives, University of Notre Dame, Notre Dame, IN, USA; Department of Anthropology, University of Notre Dame, Notre Dame, IN, USA

**Keywords:** body mass index, obesity, cold climates, metabolically healthy obese phenotype, physical activity

## Abstract

As global obesity rates continue to rise, it is important to understand the origin, role and range of human variation of body mass index (BMI) in assessing health and healthcare. A growing body of evidence suggests that BMI is a poor indicator of health across populations, and that there may be a metabolically healthy obese phenotype. Here, we review the reasons why BMI is an inadequate tool for assessing cardiometabolic health. We then suggest that cold climate adaptations may also render BMI an uninformative metric. Underlying evolutionary and environmental drivers may allow for heat conserving larger body sizes without necessarily increasing metabolic health risks. However, there may also be a potential mismatch between modern obesogenic environments and adaptations to cold climates, highlighting the need to further investigate the potential for metabolically healthy obese phenotypes among circumpolar and other populations as well as the broader meaning for metabolic health.

## INTRODUCTION

Global obesity rates continue to reach all-time highs as over 10% of the human population is living with obesity. With over 108 million children and 604 million adults living with obesity, the risks of associated metabolic health issues are on the rise globally, but especially in developing nations [1]. Risk factors associated with unhealthy weight gain include hypertension, insulin resistance, cardiovascular disease and cancer. As a result, obesity is associated with high rates of all-cause mortality [2, 3]. Obesity is also associated with an additional economic burden. Individuals living with obesity accrue medical costs estimated to be 30% higher than those considered to be at a healthy weight [4]. These increases in health care costs rise to 42.7% for individuals with obesity in the USA [5].

**Figure 1.**
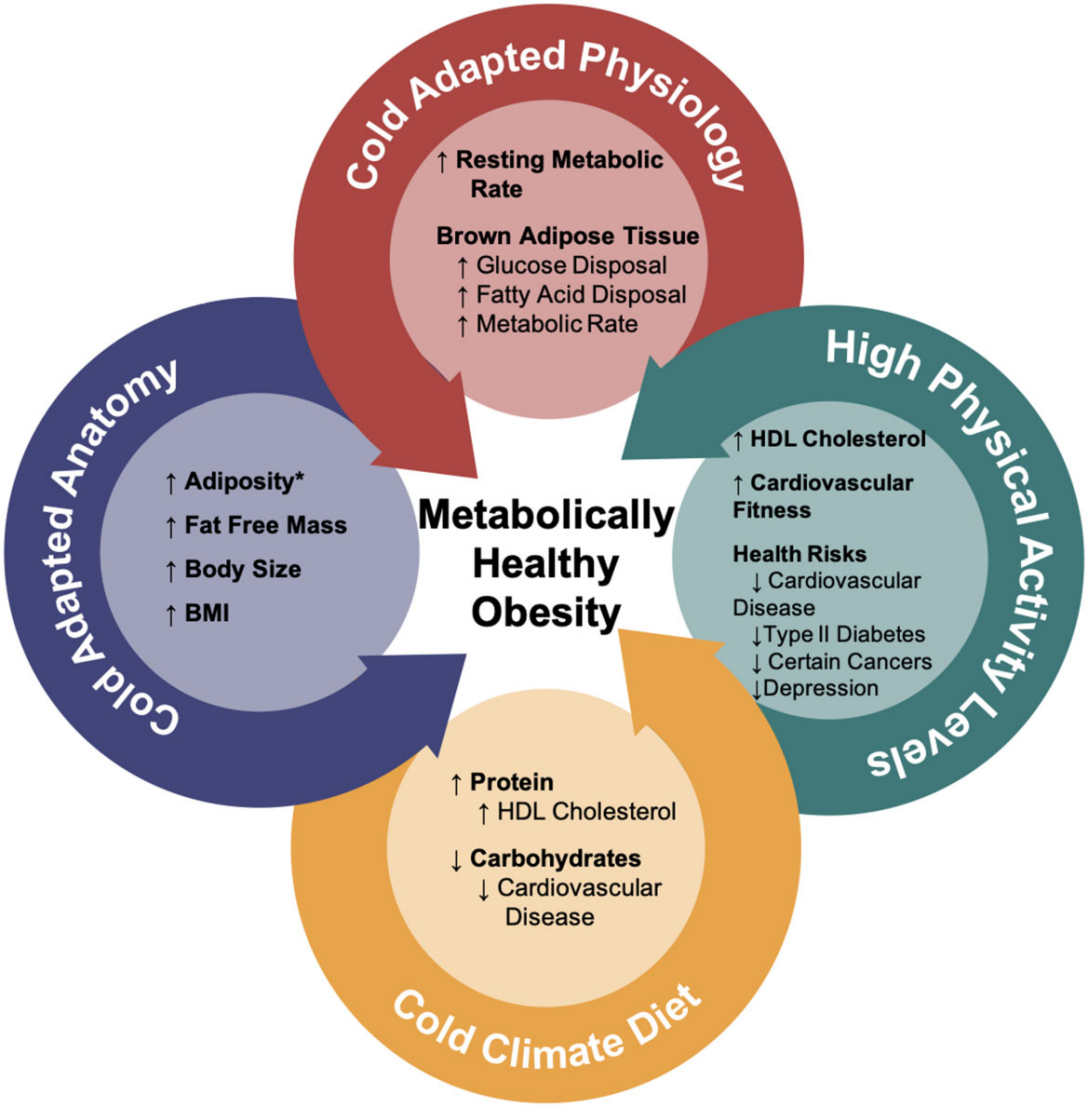
The different characteristics present among cold climate populations that may confer a metabolically healthy obese phenotype. * the relationship of greater adiposity among cold climate populations may be stronger among females rather than males [6]

The definition of obesity and the statistics used to assess health status and economic consequences rely heavily on the body mass index (BMI, kg/m^2^) metric. However, this overreliance on BMI to assess cardiometabolic health across all populations is facing greater scrutiny. Populational variation arising from evolutionary, ecological and cultural histories could significantly impact the relationship between BMI and markers of cardiometabolic health such that current BMI standards and associated comorbidities do not accurately represent all populations. Here we briefly review the development and use of BMI. We then discuss the mounting criticisms of this metric framed within the metabolically healthy obese phenotype—the ‘fit and fat’ hypothesis—a hypothesis that is still quite controversial. Finally, we suggest that cold climate populations, due to their evolutionary history, might be the most logical candidates for garnering greater evidence in support of the metabolically healthy obese phenotype (Fig. 1).

### Historical background and shortcomings of BMI

BMI was originally developed by 1800 s statistician Adolphe Quetelet [7]. Quetelet was interested in means of human anthropometric measures, believing a statistical norm fitting within a classic bell curve represented the norm for man [7, 8]. Of particular interest to Quetelet was the relationship between weight and height. From looking at French and Scottish men, he found that weight scaled best to the square of height, which he called the Quetelet Index [7, 8]. Roughly 140 years later, physiologist Ancel Keys sought a weight-to-height metric that most accurately represented body adiposity for medical applications. Keys tested various ratios using data among 7426 men from the USA, Japan, Finland, Italy and Bantu men of South Africa. Keys *et al.* determined that the Quetelet Index, then renamed Body Mass Index, best fit their data. Though Keys admitted that BMI was imperfect, he contended it was better than other ratios at determining obesity and was a simple tool that could be applied to *all* populations [8]. The word ‘all’ is emphasized here as Keys did not have global representation. BMI did not produce an accurate representation of the included Bantu population, and no women were included in this analysis—calling into question the broad applicability of BMI for identifying obesity or body adiposity. However, despite these issues, in 1985 the National Institutes of Health adopted BMI as an obesity identifier, and in 1998 revised the definition to lower the BMI thresholds for ‘overweight’ and ‘obese’ to the current categories and ranges we use today [9]. In all of this, it should be noted that Quetelet was quite explicit that his index should be used for statistical purposes and not to assess individuals or health [10].

Since the NIH adoption, BMI has become near synonymous with health and numerous comorbidities. High blood pressure, high cholesterol, type 2 diabetes, osteoarthritis, some cancers and coronary heart disease among others have all been correlated with a high BMI [11, 12]. However, one of the chief weaknesses of BMI is that it does not take body composition, particularly body adiposity versus fat-free mass, into account as adiposity levels are typically more indicative of cardiometabolic health than is any metric of overall body size [13, 14]. Some medical practitioners have recently called for the medical community to follow the Adiposity-Based Chronic Disease concept, which focuses more on obesity manifested pathophysiology such as the above-mentioned cardiometabolic risk factors rather than a crude measure of body size alone [13, 15].

As more data are collected, the more obvious it becomes that BMI does not accurately indicate obesity or metabolic risk factors similarly across different populations. For example, it has been documented that there is a disconnect between body fat percentage and BMI among Asian populations such that these populations exhibit a low BMI despite a high body fat percentage [16, 17]. Furthermore, BMI underestimates potential health risks among Asian populations, particularly type II diabetes prevalence in Japan [12, 18], and overestimates adiposity among Black populations, especially among Black women as was found in Australia [19].

### Why metabolic health is not necessarily associated with BMI

In a European study comparing metabolic health markers among individuals with obesity, 12% of participants did not present any of the metabolic syndrome symptoms such as elevated blood pressure, fasting glucose or cholesterol levels associated with having a BMI ≥ 30 [20]. In a UK study using the homeostasis model assessment of insulin resistance to define metabolic health, up to 40% of participants classified as obese were considered metabolically healthy [21]. While obesity increases the risk of metabolic syndrome over time, younger and female individuals with high BMIs were more likely to be metabolically healthy relative to their older and/or male counterparts [22].

The role of physical activity further complicates the utility of any simplistic metric to accurately assess obesity-associated risk factors. Physical activity has numerous cardiometabolic benefits that may even counteract the potentially harmful effects of a high BMI, or more accurately, high adiposity, may have on the body [23–26]. Observational studies have demonstrated that physical activity and physical fitness can attenuate the health risks of high levels of adiposity such as type II diabetes, certain cancers, depression and cardiovascular disease as well as increase the levels of healthy high-density lipoprotein cholesterol (see [27]). These studies suggest a ‘fit, but fat’ or metabolically healthy obese phenotype, in which one can exhibit a high BMI and adiposity but not suffer negative health consequences [2, 23–26, 28, 29]. However, it should be noted that some dispute the potential for a metabolically healthy obese phenotype, suggesting that current data, which are largely cross-sectional, are insufficient to support this hypothesis [30]. Studies that contest the existence of a fit, but fat phenotype often rely solely on BMI to define obesity, without consistent measure of metabolic and cardiovascular health, and these studies rarely control for physical activity levels and fitness (e.g. [30–32]). This is a common concern with a number of the large meta-analyses examining BMI and cardiometabolic risk factors as most do not incorporate measures of physical activity and/or cardiovascular fitness levels such as frequency of exercise or maximal exercise oxygen consumption—commonly known as VO_2_ max [33].

Numerous studies have found that risks associated with a high BMI are greatly reduced by high levels of physical activity and/or high levels of cardiorespiratory fitness. Cardiorespiratory fitness here is defined as the ability of the circulatory and respiratory systems to supply oxygen during sustained physical activity [34]. Data from over 4600 adults ages 20–49 who took part in the National Health and Nutrition Examination Survey revealed that roughly 17.4% of the adults classified as ‘overweight’ and 8.9% of adults classified as ‘obese’ had a high degree of cardiovascular fitness [23]. This study established that at least in this population, there is a phenotype of being ‘overweight’ or ‘obese’ but also cardiovascularly fit. It should be noted that older individuals (≥50 years) were not included in this study—ages at which there is typically a reduction in cardiovascular fitness and increase in cardiovascular disease.

Other work has looked specifically at physical activity, BMI, and health outcomes. For example, among over 43 200 women and men from the Aerobic Center Longitudinal Study in the USA with a mean age of just of over 46 years in age it was found that when adjusting for fitness levels, metabolically healthy obese (determined by body fat percentage) individuals had significantly lower cardiovascular hazard ratios relative to their non-metabolically healthy obese counterparts. Metabolic health was determined by measures of blood pressure, HDL cholesterol, triglyceride levels and fasting glucose levels. Individuals were considered metabolically healthy if only 0–1 of these metrics were outside of recommended values. Furthermore, there were no significant differences in cardiovascular health ratios between metabolically healthy obese individuals and metabolically healthy normal-fat individuals [33]. Similar results were found among over 47 200 Finnish women and men [3] and ∼40 000 women part of the Women’s Health Study [35]—regular physical activity was strongly associated with reduced all-cause mortality or coronary heart disease in these studies, respectively. Similarly, work among reindeer herders in sub-Arctic Finland found that despite 75% of the participants being overweight or having obesity, their metabolic health markers (total cholesterol, HDL cholesterol, LDL cholesterol, glucose and triglycerides) were relatively normal [36].

Among the potential reasons for the disconnect between BMI and cardiometabolic health among this sample is the high level of physical activity. Reindeer herders are highly active—they expend a mean of ∼4200 kcal/day during their annual herd roundup, which puts their total energy expenditure at a level similar to that of hot climate farming populations [37]. However, the high levels of physical activity may not be the only reason for the BMI-health disconnect. Cold climate adaptations could also impact the relationship between body size and metabolic health. All of these studies add further evidence that the metabolically healthy obese phenotype does exist, and that having obesity (at least as defined by BMI) does not necessarily mean poor metabolic health.

### Evolutionary and environmental drivers of metabolically healthy obesity

Numerous well-known cold climate adaptations that appear among circumpolar populations include a larger body size with a reduced body surface area [6, 39, 40], high resting metabolic rates [38, 41, 42], shivering thermogenesis and brown adipose tissue activity [36, 43, 44], oscillating vasoconstriction and vasodilation [45], as well as numerous cultural mitigation strategies [46]. Less well studied, however, is if cold climate adaptations may affect the relationship of body size and adiposity to biomarkers of cardiometabolic health. Notable exceptions include work done among Indigenous Siberians, examples from this work appear throughout this commentary [43, 47–52]. Below is a brief review of some cold climate adaptations that may contribute to a metabolically healthy obese phenotype.

Bergmann’s [53] ecogeographical rule predicts that body size increases with lower ambient temperature—essentially reducing body surface area while increasing metabolic heat output has been found to still hold true among human populations [39, 54, 55]. Furthermore, both fat-free mass [56] and adipose tissue are highly insulative. Laboratory studies have demonstrated that when submerged in cold water, individuals with greater body fat experienced a reduced drop in body temperature and a mitigated metabolic response relative to those with less body fat [57]. Thus, it is believed that greater body size and greater body adiposity were crucial evolutionary adaptations to cold climates—as these features would increase metabolic heat production while also reducing heat lost to a cold environment.

Early work among Inuit populations [58, 59] found that individuals in these communities tended to have larger, though leaner bodies, especially among males [6]. Furthermore, it was found that skinfold thickness measures did not correlate well with body fatness as determined by the deuterium oxide dilution method. This work indicates a large-bodied phenotype, but also revealed a high degree of physical fitness [6]. As such, the high body mass index among reindeer herders mentioned above, like North American cold climate populations, should not come as a surprise.

We now recognize that adipose tissue operates like an organ with its own suite of hormonal and regulatory functions [60–64], which means that it can also display dysfunction. Moreover, the location and distribution of body fat is a key variable affecting the relationship between body adiposity and metabolic health. Visceral fat, located around the abdominal organs, is associated with an increased risk of type II diabetes, cardiovascular disease, inflammation, hormonal imbalances and fatty liver disease relative to subcutaneous fat which is located between the skin and muscle [60, 65, 66].

Fat distribution patterns also vary between populations. Despite having lower average BMIs, East and South Asian populations tend to have greater total body fat and higher body fat percentages compared to Europeans [16, 66, 67]. Individuals of Chinese and South Asian descent have greater amounts of visceral and subcutaneous abdominal adipose tissue than Europeans with similar BMIs [68]. Africans, however, have less visceral and more subcutaneous fat relative to Europeans of similar weight and age [69, 70]. These variations in visceral fat distribution between populations explain in part why Asians, despite having lower average BMI measurements, tend to have higher rates of type II diabetes compared to Africans and Europeans [71]. As such, some have called for new diagnostic approaches to metabolic disease and obesity with a greater focus on adiposity, especially visceral adiposity, either through measures of waist-to-hip ratio or body composition [13, 15].

Cold climate populations tend to have high resting metabolic rates (RMR, kcal/day) in order to maintain core body temperature despite cold ambient temperatures [38, 42, 72, 73]. The high RMR is thought to be driven, though not yet proven, by high thyroid hormone levels [72, 74]. Though an important cold climate adaptation and one that does lead to greater energy expenditure, high RMRs may also be associated with high blood pressure. A study examining this relationship among Nigerians and African-Americans found that individuals with high RMRs did have high blood pressure independent of body size [75]. The positive association between blood pressure, particularly systolic blood pressure, and RMR was also found among three Indigenous cold climate populations in Siberia: the Sakha, Evenki and the Buryat [49]. Hypertension is common among cold climate populations and is likely the result of numerous interacting factors including high RMRs, diets high in salt, fat, and alcohol, psychosocial stress, and socioeconomic status. However, it has been suggested that increased sympathetic tone, thyroid hormone levels, oxidative stress, and/or developmental patterns (i.e. low birth weight) may be driving this causal link between high RMR and hypertension [49, 75]. These results paint an ever more complicated picture of cold climate population health and the apparent disconnect with body shape and size.

Brown adipose tissue (BAT), a type of mitochondria dense fat, has received a great deal of recent attention both for its ability to increase heat production during cold exposure and for how it may alter metabolic rate and circulating blood glucose and fatty acid levels [43, 44, 76, 77]. BAT has been identified among cold [36, 42] and temperate climate populations [72, 73] during acute cold exposure, and has been shown to increase metabolic rate from 3 to 9.5% depending on the population. For example, reindeer herders in sub-Arctic Finland trended on the higher end of that metabolic rate increase (8.7%) and preferentially metabolized fatty acids rather than glucose [80].

The evolutionary impact of BAT activity may result in more than just thermal protection by significantly altering blood glucose and fatty acid levels. In a small study conducted among seven males, acute cold exposure elicited a 63% increase in fatty acid utilization and a 588% increase in glucose utilization [81]. Among the Sakha, glucose levels, but not fatty acid levels, were positively correlated with BAT activity [43]; a similar preference for glucose utilization was also found among a population of females and males in Albany, NY [78]. Chondronikola *et al.* [82] found that the increase in metabolic rate associated with BAT was 70% fueled by free fatty acids and 30% by glucose, while others found an increase in LDL and HDL cholesterol levels [83]—indicating a high degree of variation in BAT substrate utilization. Other work has also shown that BAT activation complicated the relationship between BMI and metabolic health.

For example, a study among 260 individuals from Japan found that those with active BAT (48% of participants) had better measures of body adiposity, LDL cholesterol, HDL cholesterol and blood glucose [84]. Improved glucose metabolism has also been confirmed among a group of males with obesity who were part of a 10-day cold exposure treatment during which BAT was measured, and investigators found that glucose uptake in skeletal muscle was particularly improved during this experimental exposure [85]. Furthermore, individuals with positive BAT activity, despite having overall greater body adiposity, had lower visceral fat than individuals with lower overall body adiposity, and this lower visceral adiposity was also associated with lower insulin resistance and inflammation as well as improved indicators of fatty liver disease [86]. Recent work also shows that BAT activity may contribute to increased thermogenesis and glucose disposal in a Samoan group, a tropical population, suggesting BAT is a metabolically active trait across all human populations, irrespective of the climatic environment [87].

Though research on BAT among adult humans is still relatively new, it is clear that BAT activity can alter cardiometabolic biomarker profiles during mild cold exposure though long-term effects have not been explored. Populations who utilize BAT activity as an adaptation to cold conditions may have a broader range of healthy baseline glucose and cholesterol levels that does not necessarily indicate a cardiometabolic concern; however, this hypothesis needs to be tested. A great deal more research measuring seasonal BAT activity and associated cardiometabolic biomarker levels as well as data on cardiovascular disease mortality and diabetes incidence are needed to test this hypothesis.

Finally, cold climate diets may influence metabolic health. Traditional cold climate diets are relatively high in protein and fat while relatively low in carbohydrates, with those calories obtained from hunted, foraged, and raised/grown foods (i.e. [38, 88, 89]). Given the consumed meat tends to be from wild or semi-domesticated mammals and fish, the diets are high in the ‘good’ HDL cholesterol. Shepard and Rode [89] argued that this more traditional diet may play a role in the relatively low rates of type II diabetes and cardiovascular disease. The combined evidence of larger body sizes, higher resting metabolic rates, modulation of cardiometabolic health biomarkers by BAT and diet suggest that perhaps we should be more closely examining cold climate populations for metabolically healthy obese phenotypes as they may be more common among these populations (Fig. 1). However, many Indigenous cold climate populations today are suffering from an epidemic of poor cardiometabolic health.

### Metabolic health in circumpolar populations

Historical work among circumpolar populations revealed relatively low mortality from cardiovascular disease and incidence of diabetes as well as lower blood glucose and lipid levels [90, 91]. A disconnect between BMI, body adiposity and metabolic health has been observed among Inuit populations in Canada, Greenland and the USA. Individuals in these populations display high rates of obesity and high adiposity but exhibit lower metabolic risk indicators (glucose and blood pressure values, though cholesterol levels were rising) relative to Euro-Canadians of the same BMI [92, 93]. Young’s work among the central Canadian Arctic Inuit found that increasing rates of obesity and greater centralized adiposity were associated with higher blood pressures and cholesterol levels, though there was no change in glucose and insulin [92]. However, in a later study, Young *et al.* [93] learned that though there was evidence for worsening blood pressure and cholesterol levels with increasing rates of obesity, the worsening health metrics were more prevalent among Euro-Canadians than among Indigenous Inuit from Canada, Alaska and Greenland.

Recent drastic changes in diet, physical activity levels and cold exposure due to climate change are further driving this trend of worsening health [42, 91–95]. Rising rates of high blood pressure, smoking, and central adiposity among circumpolar populations of North America expose a large percentage of Indigenous individuals to increased risks of cardiovascular disease, especially in women [96]. In recent years, rates of diabetes mellitus were three times more prevalent in Native Americans and Native Alaskans compared to age-adjusted Americans of European descent [97]. Similar biological and behavioral risk factors were noted to contribute to increases in cardiovascular disease among Inuit populations in the USA, Canada, Russia and Scandinavia as early as the late 1990s, early 2000s and continue to be prevalent today [98]. This health transition from relatively healthy metabolic profiles to the recent rise in risk factors is associated with the large-scale industrialization of the regions, contamination of traditional foods, reduction in traditional activities and activity levels and other negative socio-economic changes [98].

Climate change may also be a contributing factor to the worsening of cold climate population health. For example, as global temperatures continue to rise and in conjunction with modern technology, cold stress is no longer quite as stressful as it once was. As such, there may be reduced pressure to maintain high resting metabolic rates among cold climate populations [38]. This reduction in resting energy expenditure along with a reduction in physical activity and a move away from relatively healthier traditional diets all likely contribute to the growing trend in obesity rates and poor cardiometabolic health. A detailed discussion on this potential and its theoretical underpinnings can be found in [94].

Increasing rates in cardiovascular disease and risks can be traced back to the harm inflicted upon Indigenous populations by European colonization, US policies and ensuing racism, assimilation and inequities [48, 92, 93, 99, 100]. Over 68% of Nunavut Inuit suffer from food insecurity, resulting from a shift away from traditional diets to high sugar and transfatty acid foods [101]. The impacts of government policies and systemic racism have long-lasting effects on health through ongoing inequities and intergenerational trauma as best described by Godfrey and colleagues [99]. Forced relocations, pollution and lack of safe access to healthy food continuously contribute to rate increases in metabolic syndrome among Indigenous populations and to greater health disparities relative to whites [99]. The climate change crisis is further exacerbating mental and physical health problems among Indigenous groups. Extreme weather events are likely to impact remote circumpolar groups earlier and more severely than white and/or non-circumpolar individuals, further reducing safe and equitable access to the foods and services needed for improved metabolic health [102].

It is also possible that we are witnessing an evolutionary mismatch in action in which there was a benefit for larger bodies and greater adiposity in cold climates without a negative effect on metabolic health. However, the predisposition for greater adiposity becomes problematic when the imposed modern environment of lower physical activity levels and highly processed foods overwhelms and negates any potential metabolic benefits of cold climate adaptations such as BAT activity and higher resting metabolic rates. This mismatch could well explain why evidence for a metabolically healthy obese phenotype is relatively rare among cold climate populations with the exception of the reindeer herders in sub-Arctic Finland, who are more physically active than other cold climate populations for whom we have data [38]. However, a great deal more work is needed to determine the occurrence of the metabolically healthy obese phenotype among cold climate populations and beyond.

## CONCLUSION

A growing body of evidence suggests that BMI is a poor indicator of metabolic health across populations but also at the individual level. This broadly applied metric cannot take into account differences in body composition, physical activity levels and fitness, nor how certain physiological features (i.e. brown adipose tissue) may affect metabolic health. Here we have reviewed the various reasons why the association between BMI and metabolic health across populations breaks down and suggest that evolutionary and environmental trajectories may drive a metabolically healthy obese phenotype. The combination of larger body size and greater adiposity for retaining heat, higher resting metabolic rates, and higher brown adipose tissue activity with the ability to modulate cardiometabolic health suggest that metabolically healthy obese phenotypes may be more frequent among cold climate populations. However, as seen with the tendency for high blood pressure among cold climate populations this relationship is complicated and likely highly dependent on sex, age and behavioral factors. Furthermore, we may be witnessing a mismatch between evolved cold climate adaptations and the modern obesogenic environment. We suggest that further research into the potential for metabolically healthy obesity among cold climate populations is not only warranted but necessary for us to understand how evolutionary trajectories have shaped the full range of human metabolic health and could better inform the ways in which individuals with obesity are treated.

## Data Availability

No new data were generated or analysed in support of this research.
